# Indirect costs and incidence of caregivers’ short-term absenteeism in Poland, 2006–2016

**DOI:** 10.1186/s12889-019-6952-5

**Published:** 2019-05-17

**Authors:** Błażej Łyszczarz

**Affiliations:** Department of Public Health, Faculty of Health Sciences, Nicolaus Copernicus University in Toruń, ul. Sandomierska 16, 85-830 Bydgoszcz, Poland

**Keywords:** Caregiving, Indirect cost, Absenteeism, Cost-of-care, Economic losses, Poland

## Abstract

**Background:**

There is a growing interest in the costs of informal care; however, the results of previous studies mostly rely on self-reported data, which is subject to numerous biases. The aim of this study is to contribute to the topic by estimating the indirect costs of short-term absenteeism associated with informal caregiving in Poland with the use of social insurance data on care absence incidence.

**Methods:**

The human capital method was used to estimate the indirect costs of caregiving from a societal perspective. The incidence of caregiving was identified based on the Social Insurance Institution’s data on absence days attributable to care provided to children and other family members. Gross domestic product (GDP) per worker was used as a proxy of labour productivity. Deterministic one-way sensitivity analysis was performed.

**Results:**

The indirect costs of short-term caregivers’ absenteeism in Poland was €306.2 million (0.116% of GDP) in 2006 and increased to €824.0 million in 2016 (0.180% of GDP). The number of care absence days grew from 5.9 million (0.45 days per worker) in 2006 to 10.6 million (0.70 days per worker) in 2016. Approximately 85% of the total costs were attributable to child care. The results of the sensitivity analysis show that the indirect costs varied from the base scenario by − 30.8 to + 15.8%.

**Conclusion:**

Informal short-term caregiving leads to substantial productivity losses in the Polish economy, and the dynamic upward trend of care absence incidence suggests that the costs of caregiving are expected to rise in the future.

**Electronic supplementary material:**

The online version of this article (10.1186/s12889-019-6952-5) contains supplementary material, which is available to authorized users.

## Background

Informal caregiving for those requiring care during sickness is a complex activity that has multidimensional consequences for both the care recipients and the carers. The former can benefit from personalized and more supportive care, resulting in improved well-being and health status [1, 2]. On the other hand, caregivers face increased psychological and physical distress and, if left unsupported, may experience health deterioration [3] as well as other negative social and economic consequences, including financial strains and work disruptions [4, 5].

Health services research in the last two decades has witnessed a growing interest in the economic consequences of caregiving, with a particular focus on the costs of informal care delivery [6–8]. These costs can be examined at an individual, household, and societal level, and recent taxonomy lists twelve caregiving cost categories grouped into three domains: employment consequences, out-of-pocket expenses, and caregiving labour [9]. Because non-institutionalized care provided by a family member or another related person is not a paid occupation, and because it is located outside of the formal economy [10], the costs of caregiving are not easy to capture [11]. The traditional approach to cost evaluation based on identifying financial flows (reimbursements, fees, remunerations) is of little use here, and this has resulted in the development of methods allowing for indirect valuation of informal care provision by attributing certain values based on opportunity cost, proxy good prices or contingent valuation, among others [12]. All of these methods have certain weaknesses, and their usefulness depends on the study context [13]. Regardless of the method used for care valuation, most of the studies on the cost of care rely on self-reported data [14]. This fact reflects the informal character of family caregiving, which is generally located beyond the scope of data collection for administrative purposes. With low accessibility to data on formal economic activity associated with family caregiving, researchers often need to rely on self-reported data, which is subject to numerous biases [15], and for this reason, caution is needed in drawing conclusions from studies based on surveys.

Numerous studies have reported on the cost of care provided informally, both in the context of particular diseases and – less often – for overall costs regardless of the condition requiring care. Of the 365 cost-of-illness studies reviewed in 2006, 75 papers estimated the cost of informal care [16]; 74 of these were disease-specific analyses, while only one study examined the cost of care for all diseases together. Despite this imbalance in the focus of cost-of-care research, several studies attempted to assess the aggregate societal cost of caregiving regardless of a disease category. A US study estimated the market value of care provided by unpaid family members and friends to ill and disabled adults at $196 billion in 1997, the amount exceeding the costs of home health care and nursing home care combined [17]. A more recent American study estimated the cost of caregivers’ short-term absenteeism in 2010 at $25.2 billion [18] or 0.168% of the country’s gross domestic product (GDP). Another study analysed the replacement cost of informal caregiving in Australia (2015 data), providing an estimated cost of $60.3 billion, equivalent to 3.8% of GDP [19].

Most of the studies concerned with the cost of caregiving focus on long-term care provided to the elderly population, because this caring process generates the highest burden, in terms of both physical and emotional constraints and economic consequences [20–22]. Less is known about the caregiving process for children; in this case, most of the economic evidence is limited to the cost of specific conditions [23–25] and the overall incidence or determinants of all-cause absenteeism with no explicit reference to cost analysis [26, 27]. There is also little evidence regarding the cost of care provided occasionally, particularly by those who remain active in the labour market and deliver care while absent from work. It should be emphasized that such short-term informal care is addressed to diverse recipients, including sick children and adults suffering from conditions with relatively short recovery prospects or from long-term disabilities and chronic diseases. In the approach used in this study, the term ‘short-term’ applies to the period caregivers devote to providing care, not to the duration of care recipient’s sickness.

Therefore, this study is concerned with the indirect costs (productivity losses) of short-term informal caregiving in Poland, and it attempts to contribute to the growing literature on the economic consequences of informal care by addressing some of the gaps and methodological issues mentioned above.

## Methods

To assess the magnitude and costs of caregivers’ short-term absenteeism, data from the Social Insurance Institution (SII), which provides care allowances for the working population in Poland, was used. Numbers of absence episodes and days associated with care provided to a relative reported by the SII were used to identify the amount of time lost from work due to caregiving. Care allowance in Poland is granted to a person who provides care for a child or other family member. The former category refers to children up to the age of 14, while the latter category includes spouses, parents, a child’s parent, stepparents, in-laws, grandparents, grandchildren, siblings and children aged > 14. Hence, the category referring to care provided to ‘others’ also includes older children because the way the data are reported does not allow them to be separated from the total number. Consequently, ‘child care’ only accounts for children up to 14 years of age. The total duration of care allowance per household is limited to 60 days for child care and 14 days for care of others per year, regardless of the number of people for whom care is provided [28]. However, the insured caregiver is able to use more absence days in a year; in such a case the caregiver does not receive allowance and is not paid for the time of absence. The data on the number of absence days and episodes used here contains both paid and unpaid absences. Therefore, it reflects the total time of work lost because of caregiving even if the allowance is not paid. Any days of care that are provided during an employee’s holiday are not reported in the data used here. Part-time employment is not accounted for in SII’s data collection; this means that a workday lost because of a part-time worker absence was counted as a whole day. Thus, an adjustment for part-time employment was used as a part of the sensitivity analysis, and it was assumed that an average part-time employee works half-time (20 h weekly).

All the data on incidence and length of absence episodes were taken from SII’s yearly reports on sickness absence in Poland [29]. As such, the analysis only accounts for short-term care episodes; however, it does not make any distinction with regard to the duration of sickness. Therefore, care for those suffering from long-term or chronic diseases is also included in the analysis, but only if the caregivers provide assistance on a short-term basis.

The human capital method (HCM), which is the most common approach to indirect costs analyses [30], was used to estimate the productivity losses of caregiving from a societal perspective. Using the HCM means that all potential production not performed by caregivers because of relatives’ morbidity was counted as lost productivity (indirect cost) [16, 31]. The HCM was chosen because, in short-term absenteeism, it is unlikely that a person absent from work would be replaced by employing another worker; thus, an alternative method of friction cost does not seem to be an appropriate choice here. The productivity losses identified referred to all diseases combined, and no information on disease-specific absence was obtainable. Average gross domestic product (GDP) per worker was used to proxy labour productivity, and it was adjusted for decreasing marginal labour productivity. According to the law of diminishing marginal productivity, each additional worker produces a decreasing increment of output, and for this reason, the output increments that would have been gained in the absence of the caregiving would have been lower for each additional employee compared to the average productivity in the economy. To account for this fact, a 0.65 correction coefficient that reflects a relationship between marginal and average productivity and approximates the output elasticity of labour in the Cobb-Douglas production function in Europe [32] was applied. The costs were expressed in Euro (€) currency using the average exchange rate from the period of 2006–2016, which is 4.07 zlotys per €.

Deterministic one-way sensitivity analysis was performed to test how variations in model parameters affect the cost estimates. The following parameter changes for the results for the year 2016 were used:

- extreme values of exchange rates from the whole period;
- ±0.05 changes in a coefficient adjusting for labour productivity (as suggested in recommendations for indirect cost estimation methods in Poland [33]); and
- an additional − 0.15 decrease in productivity of caregivers who potentially might be less efficient than workers on average^1^;
- gross value added instead of GDP as a measure of productivity;
- accounting for part-time employment; 6.2% of total employment in Poland in 2016 was part-time [36]; thus, it was assumed that this share of caregivers worked half-time.

The number of absence days was not subject to changes in the sensitivity analysis as it was based on exact numbers from insurance registries and not on estimates.

## Results

### Incidence of informal caregiving

The Social Insurance Institution (SII) reported 5944.6 thousand absence days related to care provided to a family member in 2006, and this number grew to 10,613.4 thousand in 2016, a growth of 78.5%. Most of these absence days (~ 85% on average throughout the period) were associated with care provided to children aged up to 14 years. There was evident gender-related disparity in caregivers’ absenteeism. The number of absence days for women was 3.1 times higher on average than that for men (7861.2 thousand vs. 2746.5 thousand in 2016); however, this disparity took a different direction in child and others’ care. Women dominated child caregiving with 4.6 times more unworked days than men, while in care for others, the number of days lost among men was 1.7 times higher than days lost among women. The average number of caregiving absence days grew from 0.45 days per worker in 2006 to 0.70 days per worker in 2016. Work absence associated with care provision accounted for 2.7% of the overall absence in the Polish economy in 2006, and this share increased to 3.7% in 2016 (Table 1).

**Table 1:** Incidence of caregivers’ short-term absenteeism in Poland, 2006–2016

	2006	2007	2008	2009	2010	2011	2012	2013	2014	2015	2016
Child care											
Number of absence days (thousand)	5079.6	6165.3	6772.1	7435.2	7169.0	7587.3	7471.6	7934.1	7589.8	8290.9	8938.4
Males	864.7	1046.8	1168.9	1278.8	1273.5	1318.0	1320.1	1465.9	1463.2	1585.4	1746.1
Females	4116.8	5079.8	5598.7	6155.9	5894.8	6268.5	6149.8	6466.1	6124.1	6702.1	7187.7
Average length of absence (days)	5.58	5.58	5.52	5.43	5.36	5.29	5.22	5.06	4.91	4.90	4.84
Absence days per worker	0.38	0.45	0.48	0.54	0.51	0.53	0.53	0.56	0.52	0.56	0.59
Share of total absence days^a^	2.3%	2.7%	2.7%	2.8%	2.8%	3.0%	2.9%	3.0%	2.9%	3.0%	3.1%
Others’ care											
Number of absence days (thousand)	865.0	1017.2	1191.5	1257.7	1265.7	1278.4	1263.0	1293.3	1362.1	1457.5	1675.0
Males	553.8	655.3	779.5	833.0	818.2	802.2	769.7	780.1	812.0	856.1	1000.4
Females	294.0	355.9	411.3	424.5	447.4	476.0	493.0	512.9	549.6	600.7	673.5
Average length of absence (days)	8.10	8.08	8.05	7.99	7.76	7.54	7.42	7.23	7.08	6.92	6.79
Absence days per worker	0.07	0.07	0.08	0.09	0.09	0.09	0.09	0.09	0.09	0.10	0.11
Share of total absence days^a^	0.4%	0.4%	0.5%	0.5%	0.5%	0.5%	0.5%	0.5%	0.5%	0.5%	0.6%
Total											
Number of absence days (thousand)	5944.6	7182.5	7963.6	8692.9	8434.7	8865.7	8734.6	9227.4	8951.9	9748.4	10,613.4
Males	1418.5	1702.1	1948.4	2111.8	2091.7	2120.2	2089.8	2246.0	2275.2	2441.5	2746.5
Females	4410.8	5435.7	6010.0	6580.4	6342.2	6744.5	6642.8	6979.0	6673.7	7302.8	7861.2
Absence days per worker	0.45	0.52	0.57	0.63	0.60	0.62	0.62	0.65	0.61	0.66	0.70
Share of total absence days^a^	2.7%	3.1%	3.2%	3.3%	3.3%	3.5%	3.4%	3.5%	3.4%	3.5%	3.7%

Notes: a – total absence days refers to the overall number of days associated with own and caregiving absence in Poland

The data on the distribution of absence by duration of absence show that the shortest care episodes (lasting 1–5 days) dominated and were more prevalent in child care than in others’ care. The average share of these short episodes for the whole period was 67.9% of all episodes in child care and 49.7% of episodes of others’ care. On the other hand, episodes lasting 11–14 days were much more common in care of other family members (30.1% on average) than of younger children (4.4% on average). In both groups, the share of the shortest episodes (1–5 days) increased notably during the 11-year period investigated; for child care, this proportion increased by 10.9 percentage points (from 62.3 to 73.2%), while for others’ care, it increased by as much as 16.4 percentage points (from 41.9 to 58.3%) (Table 2).

**Table 2:** Distribution of care episodes by duration of absence in Poland, 2006–2016

Duration	Number of care episodes in thousands (share in total)											Average share (2006–2016)
	2006	2007	2008	2009	2010	2011	2012	2013	2014	2015	2016	
Child care												
1–5 days	567.0 (62.3%)	689.9 (62.5%)	789.2 (64.3%)	903.9 (66.0%)	895.4 (66.9%)	974.9 (67.9%)	989.7 (69.1%)	1106.2 (70.5%)	1112.1 (71.9%)	1223.0 (72.3%)	1353.2 (73.2%)	67.9%
6–10 days	274.0 (30.1%)	334.0 (30.2%)	346.3 (28.2%)	368.5 (26.9%)	346.0 (25.9%)	360.2 (25.1%)	341.0 (23.8%)	365.0 (23.3%)	345.5 (22.3%)	370.9 (21.9%)	390.1 (21.1%)	25.4%
11–14 days	46.9 (5.2%)	55.1 (5.0%)	61.2 (5.0%)	63.8 (4.7%)	64.0 (4.8%)	64.6 (4.5%)	64.2 (4.5%)	62.8 (4.0%)	58.1 (3.8%)	63.4 (3.7%)	67.3 (3.6%)	4.4%
15+ days	21.3 (2.3%)	25.1 (2.3%)	30.5 (2.5%)	32.5 (2.4%)	32.4 (2.4%)	35.0 (2.4%)	35.9 (2.5%)	34.1 (2.2%)	29.5 (1.9%)	33.9 (2.0%)	36.7 (2.0%)	2.3%
Unidentified	0.5 (0.1%)	0.3 (0.0%)	0.1 (0.0%)	0.1 (0.0%)	0.0 (0.0%)	0.3 (0.0%)	0.6 (0.0%)	0.6 (0.0%)	0.7 (0.0%)	0.8 (0.0%)	1.0 (0.1%)	0.0%
Others’ care												
1–5 days	44.7 (41.9%)	53.8 (42.7%)	64.6 (43.7%)	71.4 (45.3%)	78.0 (47.8%)	84.7 (50.0%)	87.7 (51.5%)	95.3 (53.3%)	105.9 (55.1%)	119.7 (56.8%)	143.8 (58.3%)	49.7%
6–10 days	25.3 (23.7%)	28.4 (22.6%)	32.1 (21.7%)	31.5 (20.0%)	30.2 (18.5%)	30.3 (17.9%)	28.7 (16.9%)	29.5 (16.5%)	29.7 (15.4%)	31.1 (14.8%)	34.6 (14.0%)	18.4%
11–14 days	34.9 (32.7%)	41.5 (33.0%)	48.6 (32.9%)	51.9 (33.0%)	52.6 (32.2%)	51.5 (30.4%)	50.3 (29.6%)	50.1 (28.0%)	52.5 (27.3%)	55.1 (26.2%)	62.8 (25.5%)	30.1%
15+ days	1.8 (1.7%)	2.2 (1.7%)	2.6 (1.8%)	2.7 (1.7%)	2.4 (1.5%)	2.9 (1.7%)	3.4 (2.0%)	3.8 (2.1%)	4.1 (2.1%)	4.6 (2.2%)	5.2 (2.1%)	1.9%
Unidentified	0.0 (0.0%)	0.0 (0.0%)	0.0 (0.0%)	0.0 (0.0%)	0.0 (0.0%)	0.1 (0.1%)	0.1 (0.1%)	0.1 (0.1%)	0.1 (0.1%)	0.1 (0.0%)	0.1 (0.0%)	0.0%

Interestingly, there was a notable gender difference in the structure of care episodes depending on their duration in others’ care (similar data for child care were not obtainable). The shortest episodes (1–5 days) were mostly secured by women, who used 92.8 thousand care episodes in 2016, while the corresponding number for men was 50.9 thousand. On the other hand, in the care episodes lasting 6–10 days, 11–14 days and 15+ days, men were those for whom the number of care certificates was higher (Additional file 1). This pattern of gender difference was consistent over time.

**Fig. 1:**
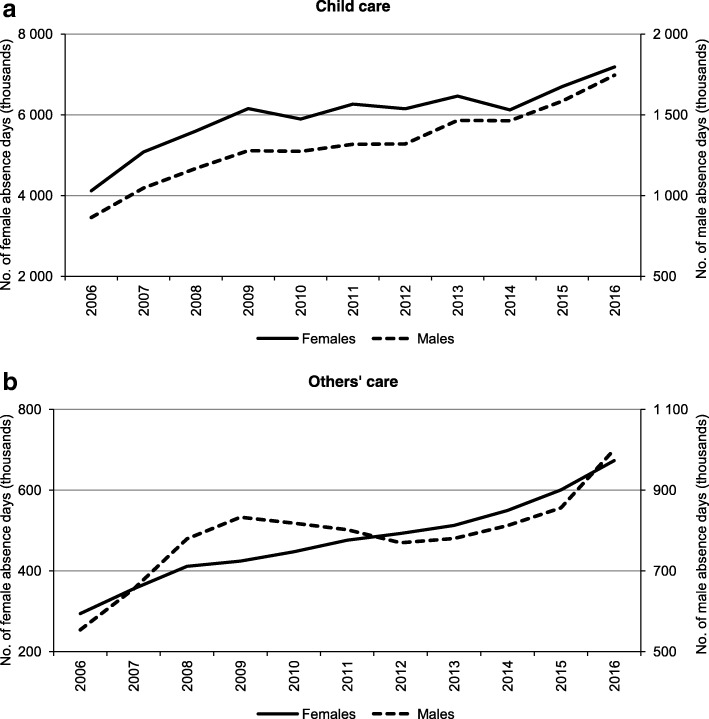
Dynamics of female and male absence days associated with caregiving provided to children (**a**) and others (**b**) in Poland, 2006–2016. Notes: The total number of female and male absence days does not add up to the total numbers in Table 1 because in the case of some absence episodes, the gender of a caregiver was not identified. This was a case of 0.3% of absence days on average

The dynamics of absence days varied depending on the caregiver’s gender and on whether a child or another person was subject to care. In child care, periods of 2006–2009 and 2015–2016 exhibited a dynamic growth of absence days for caregivers of both genders. For the years 2010–2014, though women’s absence remained fairly stable, men’s absenteeism increased perceptibly. On the other hand, in others’ care, women’s absenteeism increased each year, while in the case of care provided by men, the period of 2010–2012 shows a declining number of unworked days (Fig. 1).

**Fig. 2:**
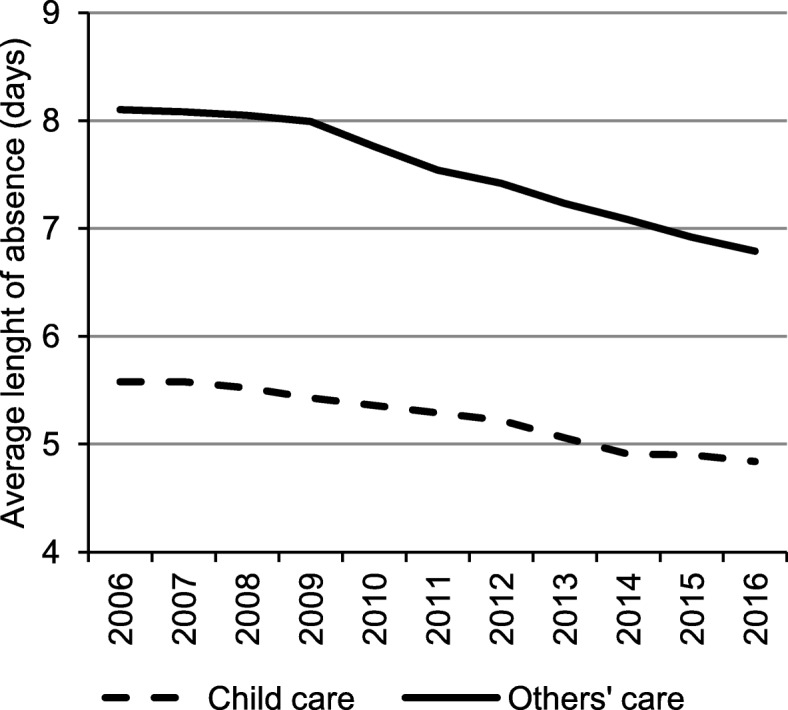
Average length of absence associated with caregiving provided to children and others in Poland, 2006–2016

The average duration of child caregiving absence was 5.58 days in 2006 and declined to 4.84 days in 2016. The absence related to adult caregiving lasted longer than that related to child caregiving: 8.10 days in 2006 and 6.79 days in 2016 on average. In both cases, the data show a continuous decline in the duration of absence (Fig. 2).

### Indirect costs

The indirect costs of caregivers’ short-term absenteeism in Poland were €306.2 million in 2006, and they increased to €824.1 million in 2016. To account for economic growth, the costs were expressed as a share of GDP; the productivity losses due to care provision accounted for 0.116% of GDP in 2006, and this share increased to 0.180% in 2016, more than a 50% rise. The total cost grew year by year, apart from 2010 and 2014 (in nominal values) and 2012 (in GDP-related terms). Care provided to children up to 14 years of age generated 84.2–86.0% of the total costs depending on year, while the remaining 14.0–15.8% resulted from care to other family members. The cost per episode of care was €301.3 in 2006 and increased to €393.4 in 2016, and it was considerably lower for child care (€287.6–€375.5) than for others’ care (€417.6–€527.6). The productivity losses due to informal caregiving translated to a country’s labour market level as a cost of €23.3 per worker in 2006 and €54.3 per worker in 2016 (Table 3).

**Table 3:** Indirect cost of caregivers’ short-term absenteeism in Poland, 2006–2016

	2006	2007	2008	2009	2010	2011	2012	2013	2014	2015	2016
Child care											
Indirect costs (€)	261,666,265	337,107,271	393,384,881	467,499,815	463,847,195	529,582,650	544,662,362	587,474,317	572,807,535	637,826,690	693,999,121
% of GDP	0.100	0.115	0.124	0.139	0.131	0.138	0.136	0.144	0.136	0.144	0.152
Cost per care episode (€)	287.6	305.2	320.5	341.5	346.7	369.0	380.5	374.5	370.5	377.0	375.5
Cost per worker (€)	19.8	24.5	28.0	33.9	32.9	37.2	38.4	41.2	39.3	43.0	45.7
Others’ care											
Indirect costs (€)	44,558,886	55,618,626	69,213,108	79,079,852	81,893,067	89,230,485	92,069,779	95,761,401	102,798,643	112,126,838	130,051,075
% of GDP	0.017	0.019	0.022	0.023	0.023	0.023	0.023	0.024	0.024	0.025	0.028
Cost per care episode (€)	417.6	441.8	468.0	502.1	501.8	526.4	541.0	535.6	534.6	532.4	527.6
Cost per worker (€)	3.4	4.0	4.9	5.7	5.8	6.3	6.5	6.7	7.1	7.6	8.6
Total											
Indirect costs (€)	306,225,151	392,725,897	462,597,989	546,579,667	545,740,261	618,813,135	636,732,142	683,235,719	675,606,179	749,953,528	824,050,196
% of GDP	0.116	0.135	0.146	0.162	0.154	0.161	0.159	0.168	0.160	0.170	0.180
Cost per care episode (€)	301.3	319.2	336.4	358.1	363.6	385.7	397.6	391.0	388.7	394.2	393.4
Cost per worker (€)	23.2	28.5	33.0	39.7	38.7	43.5	44.9	48.0	46.4	50.6	54.3

Notes: *GDP* Gross domestic product, *€* Euro

### Sensitivity analysis

Table 4 reports the results of a one-way sensitivity analysis of indirect costs associated with informal caregiving in Poland; for the sake of brevity, the analysis was only conducted for the year 2016. Using extreme values of exchange rate from the whole period varied the estimates from − 6.8 to + 15.8%. Varying the value of the coefficient adjusting for decreasing labour productivity by ±0.05 and accounting for potential lower caregivers’ productivity resulted in changes in productivity losses ranging from − 30.8 to + 7.7%. When gross value added was used as a productivity measure, the costs were 11.5% lower than in the base scenario. Accounting for part-time employment decreased indirect costs by 3.1% (Table 4).

**Table 4:** Sensitivity analysis for indirect cost of cost of caregivers’ short-term absenteeism in Poland (2016) according to varied assumptions for model parameters

	Indirect cost (€)	Change from base scenario
Base scenario (BS)	824,050,196	–
Exchange rate (BS: 4.07)		
3.51	954,311,539	15.8%
4.36	768,247,360	−6.8%
Coefficient to adjust for decreasing marginal labour productivity (BS: 0.65)		
0.45	570,496,289	−30.8%
0.70	887,438,672	7.7%
Productivity measure (BS: gross domestic product)		
Gross value added	729,355,474	−11.5%
Part-time employment adjustment	798,504,639	−3.1%

## Discussion

This is the first study to use social insurance data to assess the indirect costs associated with informal caregivers’ short-term absenteeism in Poland. Using the HCM and the societal perspective, the study estimated the economic burden of short-term caregiving in Poland for the 11-year period (2006–2016). The results show that the productivity losses attributable to the provision of short-term care were €306.2 million in 2006 and more than doubled, reaching €824.1 million in 2016. This increase was much higher than of the increase in GDP; the dynamics of the economy’s output grew by 74% in the period investigated, while the indirect costs of absence associated with care increased by 169%. This cost escalation reflects a growing number of caregivers’ absence days throughout the period and was not outweighed by the diminishing duration of the average absence episode.

Notably, the dynamic increase in caregiving incidence and costs observed in Poland cannot be explained by epidemiological patterns or demographic situation as no dramatic changes that could increase absence were observed in these areas. Consider the demography trends of the two groups with potentially the greatest care needs, namely, the young and the elderly populations: although the share of the population aged 65+ years increased by 3% in the period investigated, this rise was accompanied by a drop of similar magnitude (− 2.6%) in the population aged 0–18 years (see Additional file 2 for details). Considering that it was the youngest population that generated the vast majority of the short-term absence, it seems that the overall demographic factors in these two groups do not explain the nearly two-fold increase in the number of absence days. Thus, it is possible that factors other than health or demography may influence the frequency of caregiving; social insurance arrangements or labour market situations could potentially play a role. Of these two, the former remained fairly stable across the period; on the other hand, the labour market situation improved notably, with the unemployment rate dropping from 18% in 2006 to 8.2% in 2016. A more favourable labour market situation for employees could have resulted in a greater inclination to use care allowances because workers were less afraid of job loss. This mechanism has not been investigated in the area of caregivers’ absenteeism; however, previous studies on own absence suggest such a relationship [37–39].

Few studies have estimated the indirect costs of informal caregiving, and none of them have used a methodological approach similar to the one adopted here, which accounts for unworked days based on insurance data and is not based on potential economic losses or replacement costs. The American study from 2006 reports that the cost of absenteeism for employers was $7.0 billion, which translated to an equivalent of $441 per employed caregiver [40]; however, according to a recent review, these estimates should be treated with caution because the study makes debatable assumptions in carrying out the analysis [14]. The other research based on 2010 data estimates the cost of caregivers’ absenteeism for the US economy at $25.2 billion [18], or 0.168% of the country’s GDP. On the other hand, the opportunity costs of informal care for elderly Americans in the period 2011–12 amounted to $522 billion annually, while the cost of replacing this care varied from $221 billion to $642 billion depending on whether the care would be delivered by unskilled or skilled workers [41]. A recent Australian study estimates the replacement cost of informal caregiving in 2015 at $60.3 billion, equivalent to 3.8% of GDP [19].

This variation in the estimates of caregiving costs shows that comparability of results from a range of studies is limited because the estimates critically depend on the studies’ settings. The inclusion of such categories as employees’ replacement costs, workday interruptions or unpaid leave in some studies elevates costs compared to the cost reported in studies assessing the impact of absenteeism alone. Moreover, relying on administrative data, as in this study, restricts analysis to the productivity losses associated with absence from formal work and does not allow the inclusion of cost categories that are not routinely reported for formal purposes, such as presenteeism or the value of housekeeping services undone. Additionally, the way productivity losses are valued (opportunity costs vs. market wages or per worker productivity) affects the estimates. For these reasons, the estimates from the present study are hardly comparable with others’ findings. However, the results of the American study, which estimates the costs of absenteeism at $25.2 billion [18], are of similar magnitude in GDP-related terms (0.168% of US’s GDP in 2010) to estimates from this study (0.154% of Polish GDP in the same year).

The results of the present study emphasize complex gender-related disparities in informal care provision. Overall, women lost approximately three times more work days to caregiving than did men. However, this difference was mainly due to the disparity in caring for younger children, while men dominated care for others (adults and older children). The higher incidence of child care among women possibly reflects both an earnings gap between men and women and differences in the distribution of traditional societal roles associated with gender. Because in Poland absence associated with caregiving is subject to a 20% reduction in the employee’s earnings, the economic loss for a family caring for a child is lower when the parent receiving the lower salary provides care. Women in Poland receive 7.2% lower gross hourly earnings than men do (data for 2016) [42] and, for this reason, the cost of care for a household is on average lower when the woman provides care. Additionally, the distribution of traditional social roles attributes more care responsibilities to women [43], and this probably strengthens the gender-related earnings gap effect. On the other hand, though men are those who provide more care days to other family members, a closer inspection of this category shows a more complicated gender-related pattern of caregiving. Short care episodes (1–5 days) are mostly secured by women, while in long-lasting episodes (6 or more days), men dominate caregiving, as shown in the above results (see Additional file 1 for details). It is possible that short episodes are more often those involving older children (aged > 14) in which case women are more likely to deliver care due to the reasons explained above. On the other hand, men’s dominant role in longer care episodes perhaps reflects higher female morbidity and the fact that, in most cases, the only person able to provide care for a sick woman is her husband. In this case, there is usually no choice regarding the person who provides care, and because women are more frequently sick, informal care is provided by their male spouses.

### Limitations of the study

This study has the following limitations. Firstly, the estimates only provide evidence on the productivity losses associated with absences that were registered in the social insurance system. Thus, unrecorded care episodes (e.g. provided by those using holiday instead of formal absence) are not included and this fact underestimates the real burden. Secondly, the present study does not show the overall indirect costs of caregiving in Poland; it is limited to short-term absenteeism only, and the burden of long-term or permanent caregiving translating to economic inactivity is not investigated here. Thirdly, economic losses due to housekeeping activities undone and to presenteeism of caregivers are not included because of data unavailability, and this also biases the results downwards. Finally, these estimates do not allow the identification of the costs attributable to particular diseases; the SII does not collect data on diagnoses for caregivers’ absenteeism as it does in own sickness absenteeism, where ICD-10 codes are attributed to each absence episode.

## Conclusions

In conclusion, this study estimates the indirect costs of informal caregivers’ short-term absenteeism in Poland for the period from 2006 to 2016. Using the data on caregiving incidence from the social insurance system it has been shown that the costs of productivity losses associated with care provision for family members increased from €306.2 million in 2006 to €824.1 million in 2016. The study shows that both the incidence and the costs of caregiving in Poland are growing rapidly, and their dynamics exceed the growth rates of the economy and the dynamics of own sickness absence. The study also confirms the dominant role of women in short-term caregiving, which has been confirmed previously in several studies concerned with long-term care.

## Additional files

Additional file 1: Distribution of care episodes by duration of absence and gender of caregiver in others’ care in Poland, 2006–2016. (PDF 97 kb)

Additional file 2: Change in selected population age structure measures during the period of the study in Poland. (PDF 95 kb)

## Abbreviations

BS: Base scenario
GDP: Gross domestic product
HCM: Human capital method
SII: Social Insurance Institution
US: United States

## Footnotes

1 To the best of the author’s knowledge, estimates of productivity decrease in the general population of caregivers in short-time absence are not available. Although a growing body of disease-specific evidence exists (e.g., for advanced cancer [34] or post-stroke spasticity [35]), the applicability of these estimates in the context of short-term absenteeism is questionable. Therefore, the arbitrary change of − 0.15 was used

## Authors’ information

Błażej Łyszczarz holds a PhD in Economics and is currently Assistant Professor in the Department of Public Health, Faculty of Health Sciences, Nicolaus Copernicus University in Toruń, Poland. He is also a member of the Scientific Council at the Institute of Innovation and Responsible Development INNOWO.
