# Supplementary material for: Indirect costs and incidence of caregivers’ short-term absenteeism in Poland, 2006–2016
Source: BMC Public Health. 2019 May 17;19:598. doi: 10.1186/s12889-019-6952-5 (PMC6525462; doi:10.1186/s12889-019-6952-5)
Supplement: Supplementary file 1 — Distribution of care episodes by duration of absence and gender of caregiver in others’ care in Poland, 2006–2016. (PDF 97 kb) [file 12889_2019_6952_MOESM1_ESM.pdf]

Table A1. Distribution of care episodes by duration of absence and gender of caregiver in others' care in Poland, 2006-2016

**Duration**	**Number of episodes in thousands**										
	**2006**	**2007**	**2008**	**2009**	**2010**	**2011**	**2012**	**2013**	**2014**	**2015**	**2016**
**Males**											
1-5 days	18.5	22.3	27.4	30.1	30.9	32.2	32.1	34.1	36.7	41.9	50.9
6-10 days	16.8	18.5	21.1	20.8	19.2	18.4	16.7	16.9	16.6	17.0	19.2
11-14 days	24.3	29.5	35.3	38.6	38.8	37.4	35.7	35.6	37.5	39.0	45.4
15+ days	1.2	1.4	1.6	1.7	1.5	1.8	2.2	2.5	2.6	3.0	3.4
**Females**											
1-5 days	25.3	31.2	37.2	41.3	47.1	52.5	55.6	61.2	69.2	77.8	92.8
6-10 days	8.1	9.7	11.0	10.7	11.0	11.9	12.0	12.6	13.1	14.4	15.3
11-14 days	9.8	11.8	13.2	13.3	13.8	14.1	14.6	14.5	14.9	16.1	17.4
15+ days	0.6	0.8	1.0	1.0	0.9	1.1	1.2	1.3	1.5	1.6	1.8

Source: [29].
