# Supplementary material for: Indirect costs and incidence of caregivers’ short-term absenteeism in Poland, 2006–2016
Source: BMC Public Health. 2019 May 17;19:598. doi: 10.1186/s12889-019-6952-5 (PMC6525462; doi:10.1186/s12889-019-6952-5)
Supplement: Supplementary file 2 — Change in selected population age structure measures during the period of the study in Poland. (PDF 95 kb) [file 12889_2019_6952_MOESM2_ESM.pdf]

Table A2. Change in selected population age structure measures during the period of the study in Poland

Number in thousands (share of total population)			
**Age group**	**2006**	**2016**	**2016-2006 difference**
0-18 years	8,229.5 (22.1%)	7,286.4 (19.0%)	–943.1 (–2.6 p.p.)
65 and more years	5,116.5 (13.3%)	6,303.4 (16.4%)	1,186.9 (+3.0 p.p.)
Total population	38,125.5 (100.0%)	38,433.0 (100.0%)	307.5 (-)

Notes: p.p. – percentage point. Source: own calculation based on Central Statistical Office of Poland website (table 'Stan i struktura ludności według wieku w latach 1989-2016' [The condition and structure of the population by age in 1989-2016]; http://stat.gov.pl/obszary-tematyczne/ludnosc/ludnosc/struktura-ludnosci,16,1.html.
